# Magnetic levitation-based electromagnetic energy harvesting: a semi-analytical non-linear model for energy transduction

**DOI:** 10.1038/srep18579

**Published:** 2016-01-04

**Authors:** Marco P. Soares dos Santos, Jorge A. F. Ferreira, José A. O. Simões, Ricardo Pascoal, João Torrão, Xiaozheng Xue, Edward P. Furlani

**Affiliations:** 1 Centre for Mechanical Technology & Automation, TEMA, University of Aveiro, 3810-193 Aveiro, Portugal; 2 Department of Mechanical Engineering, University of Aveiro, 3810-193 Aveiro, Portugal; 3 Institute of Electronics and Informatics Engineering of Aveiro, IEETA, 3810-193 Aveiro, Portugal; 4 Department of Chemical and Biological Engineering, University at Buffalo, SUNY, Buffalo, NY, US; 5 Department of Electrical Engineering, University at Buffalo, SUNY, Buffalo, NY, US

## Abstract

Magnetic levitation has been used to implement low-cost and maintenance-free electromagnetic energy harvesting. The ability of levitation-based harvesting systems to operate autonomously for long periods of time makes them well-suited for self-powering a broad range of technologies. In this paper, a combined theoretical and experimental study is presented of a harvester configuration that utilizes the motion of a levitated hard-magnetic element to generate electrical power. A semi-analytical, non-linear model is introduced that enables accurate and efficient analysis of energy transduction. The model predicts the transient and steady-state response of the harvester a function of its motion (amplitude and frequency) and load impedance. Very good agreement is obtained between simulation and experiment with energy errors lower than 14.15% (mean absolute percentage error of 6.02%) and cross-correlations higher than 86%. The model provides unique insight into fundamental mechanisms of energy transduction and enables the geometric optimization of harvesters prior to fabrication and the rational design of intelligent energy harvesters.

Motion-driven electromagnetic energy harvesting systems have been used to provide self-powering for a wide range of technologies, such as remote sensors and actuators, mobile electronics, wearable devices and biomedical implants^1^,^2^,^3^,^4^,^5^,^6^,^7^. When dominant excitations are known a priori in a constrained range, the harvesters’ characteristics can be optimized prior to fabrication using accurate models. For unknown, broadband and time-varying vibration spectra, new tunable mechanisms and broadband harvesting designs have been proposed^8^,^9^,^10^,^11^,^12^,^13^,^14^. However, these harvesters are usually complex and their use is often impractical due to dimensional constraints. Besides, the tuning mechanisms must be subject to constraints and, thus, practical applications require geometric optimization. Moreover, broadband harvesters cannot ensure optimal performances if intelligent control is not used to optimize the adaptive mechanism^15^,^16^. Consequently, modelling of the energy transduction is essential for design optimization prior to fabrication, as well as to fulfil demanding adaptability requirements. However, such modeling is problematic because of the highly non-linear behavior of most harvesters.

Magnetic levitation has been used to implement low-cost and maintenance-free electromagnetic energy harvesters, with the ability to operate autonomously with stable performance for long periods of time^17^,^18^,^19^. Their non-complex design is effective in many applications involving severe dimensional constraints^19^. Besides, intelligent control algorithms can be developed to control the position of their components according to the excitations’ characteristics (for example, amplitude and frequency). Geometric optimization prior to fabrication and adaptive positional control of components cannot be accomplished using linear system models because they are not sufficient to adequately predict levitation-based energy harvesting, as such systems exhibit highly nonlinear behavior^20^,^21^. The Finite Element Method (FEM) has been used to solve the differential equations that govern the dynamics of these systems, taking into account effects such as the magnetic levitation forces between magnets and magnetic field (MF) distributions, etc.^21^,^22^,^23^,^24^. A combined approach using FEM and analytical or semi-analytical modeling has also been proposed^22^,^24^. However, the computational cost of FEM analysis is usually much greater than that of semi-analytical methods^21^, cumbersome and often impractical for system optimization. In contrast, analytical analysis readily enables complexity minimization and accuracy maximization of the computation^21^,^25^,^26^. Several analytical and semi-analytical non-linear models have been developed for modeling magnetic levitation-based energy harvesting systems^20^,^21^,^24^. So far, modeling of harvester architectures with mechanical friction has been conducted by either identifying models that are only valid for specific experimental setups (i.e. those that include mechanical and/or electrical damping identification) and, hence, not suitable for design optimization; or disregarding either the electric or the mechanical behavior; or neglecting the inductive effects of coil(s) on energy harvesting, including highly non-linear effects associated with multilayered coil(s)^20^,^21^,^22^,^23^,^24^. Some phenomena occurring in these harvesting systems have also been modeled using semi-analytical techniques for diamagnetic levitation systems^27^,^28^,^29^,^30^. Their inherent nonlinearities have not yet been observed in detail due to their insufficient levitation gaps. Besides, many applications require self-powering technology in which levitation must be stable for a broad range of orientations (with respect to the acceleration of gravity) and for unconstrained motion amplitudes of the moving magnet^7^,^17^, which is hard to accomplish by diamagnetic levitation. Moreover, no configuration has been developed to allow motion of the levitating magnet along most of the harvester length using this levitation method^27^,^28^,^29^,^30^. Hence, the potential of harvester architectures using tight-fit containers with very low friction contact must be further explored. To our best knowledge, no models have been demonstrated with the following functionalities: (a) use only analytical or semi-analytical equations to accurately predict both electrical and mechanical behavior; (b) take into account the main nonlinearities of forces that oppose magnet motion, including those due to mechanical contact between container and levitated magnet; (c) have been experimentally validated with different motional excitations (amplitude and frequency) and loads; and (d) are well-suited to be used in geometric optimization of the harvester and in intelligent harvesting for unconstrained motion amplitudes and arbitrary orientations of the harvester over a broad range of frequencies. In this paper, we introduce and demonstrate for the first time a validated semi-analytical model that addresses all these considerations.

## Semi-analytical Model

**Figure 1:**
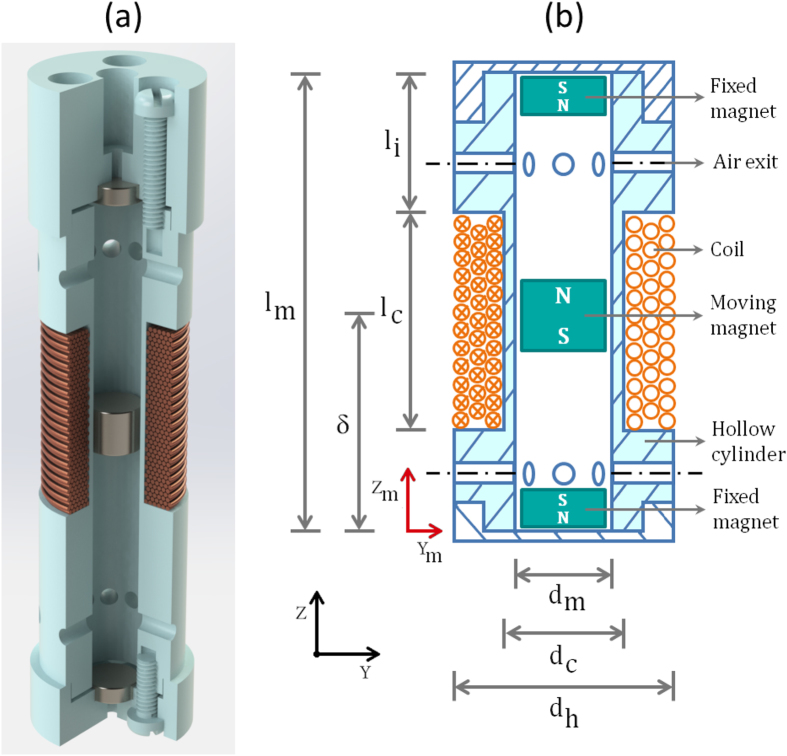
Section-views of the levitation-based harvester.

We have developed a semi-analytical model for predicting the behavior of compact magnetic levitation-based harvesters of the form shown in Fig. 1(a). These comprise a hollow cylindrical structure that houses three disc-type cylindrical permanent magnets. A portion of the cylinder is wrapped in a multilayered coil. Two of the magnets are attached to the end extremities of the container. The third magnet moves within the container between the fixed magnets and experiences a repulsive (levitation) force from each magnet. The coil is formed by winding enamelled wire around the outer surface of the container.

The MF distribution of the cylindrical magnet can be predicted using a number of analytical and semi-analytical methods^31^,^32^,^33^,^34^. In this work, we used the “equivalent” surface current model^31^ and discretize the magnet into a finite set of current loop elements. We then superimpose the MFs of the constituent current loops to obtain the magnetic field of the magnet^25^,^26^. Equations (1, 2, 3, 4, 5, 6) are used to compute the axial component of the MF, 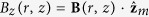, as a function of radial and axial distances (*r* and *z*) to the center of the moving magnet.


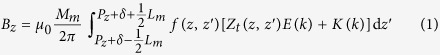



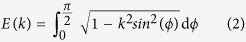



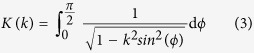



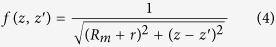



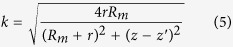



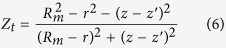


In these equations, 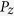 and *δ* are as defined below; 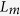, 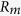 and 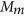 are the length, radius and saturation magnetization of the moving magnet, respectively; 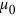 is the free-space magnetic permeability; and 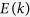 and 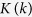 are complete elliptic integrals of the first and second kind. The use of analytical analysis to compute 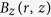 is superior to the more commonly used numerical field analysis, both in terms of accuracy and computational efficiency^25^,^26^.

The Maxwell-Faraday equation can be used to model the electromotive force induced in the coil by the time-varying magnetic flux that permeates it. One can obtain an approximate solution for predicting voltage harvesting by considering the coil as a set of single circular turns (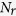 turns of the coil in the radial direction for each *z*; 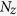 turns of the coil in the axial direction for each *r*) and a 3D surface bounded by a closed contour defined by each of these turns. Using the Kelvin-Stokes theorem, and assuming that the spatial distribution of turns along the coil is uniform, one obtain the following Equation for computing the output voltage *V* as the magnet moves within the container.


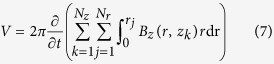


In this equation, 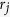 is the radius *r* of the *j*’th turn; 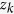 is the distance *z* of layer *k*; and *t* is the integration sample time. The voltage *V* induces a current *I* in each turn of the coil through a circuit loop composed of the impedances of the harvester and load, as expressed by Eq. (8). The load is assumed to be purely resistive 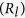 and only the coil resistance 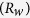 and inductance 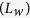 are considered^21^.


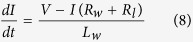


The induced current *I* gives rise to a magnetic force (Lorentz force) that opposes the motion of the moving magnet, as given by:


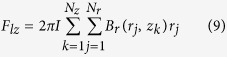


The magnetic force between two magnets as a function of their axial separation was computed by taking the derivative of their interaction energy with respect to the distance between them^35^,^36^. Equation (10) was used to compute the axial force 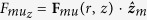 between the moving magnet and the fixed magnet located at the top of the harvester,


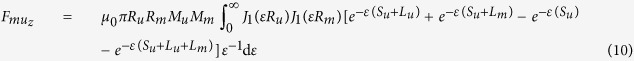


where 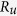 is the radius, 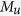 is the saturation magnetization and 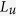 is the length, all pertaining to the fixed magnet at the top. 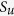 is the distance between the moving magnet and the fixed magnets at the top and 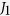 is the 1st-order Bessel function. Equation (10) can also be used to compute the force 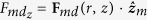 between the moving magnet and the fixed magnet located at the bottom, but using 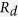, 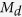, 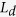 and 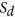. The assumption that the magnets are coaxially positioned is taken into account.

For any global coordinate system 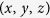, the position 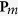 and orientation of 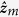 of the moving magnet can be computed by establishing the forward kinematics for translations 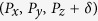 and rotations 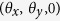 of the container about a (fixed) space system reference frame (*δ* is the distance between 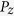 and the center of mass of the moving magnet, as shown in Fig. 1(b)). Extrinsic Euler angles 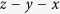 were used to define a geometric matrix transformation **Q** that expresses 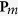 and the orientation of 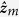.


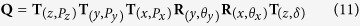



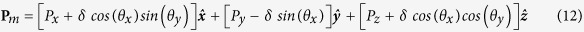



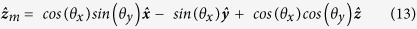


where 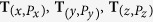 are the transformation matrices for translating 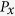, 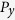 and 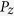 in 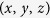; and 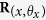 and 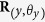 are the transformation matrices for rotating 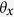 and 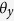 around 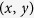. **Q** was defined considering that the influence of rotations around 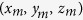 and 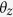 is negligible. One can use Eqs (12) and (13) to find the axial acceleration 
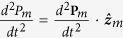
 of the moving magnet. After simplification, and considering 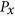, 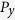, 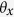 and 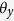 to be constant, the following algebraic equation was obtained:


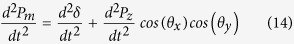


The axial component of the gravitational force 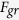 is:


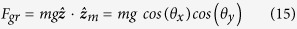


where *m* is the mass of the moving magnet and *g* is the acceleration due to gravity. The friction force 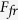 between the moving magnet and the container’s inner surface was modelled by using the Karnopp friction model^37^:


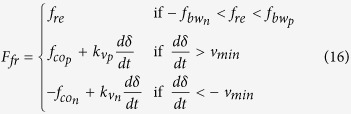



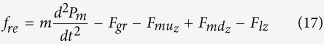


This friction model takes into account the following effects: (i) different viscous friction coefficients for negative 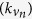 and positive 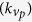 speeds 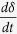; (ii) different break-away forces for negative 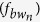 and positive 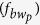 speeds; (iii) different Coulomb forces for negative 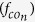 and positive 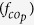 speeds; and (iv) a low speed region 
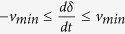
 , where one can consider 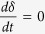. These effects can emerge due to processes used to fabricate the container.

Two differential equations are required to model the interplay between the electrical and mechanical dynamics of the moving magnet. The electrical behavior is governed by Eq. (8); the motion of the moving magnet is given by:


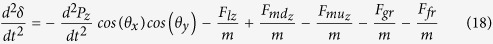


## Results

A harvester was implemented with the characteristics described in Table 1 (Fig. 1(b)). Two resistive loads were chosen: 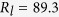 kΩ, for analyzing a quasi open-circuit voltage; and 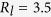 kΩ, for maximizing the power transfer to the load. To accurately analyze the nonlinearities on energy transduction of this harvester architecture, open-load conditions were discarded, such that the current dynamics could be computed for consequent analysis of friction on physical contact between the moving magnet and the container. Neodymium magnets were chosen to provide a substantial magnetic flux 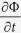 through the coils, where 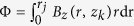. The coils were formed using enamelled copper with a very small diameter to accommodate a large number of turns. The coil was positioned so that the moving magnet is surrounded by the coil when the harvester is stationary, i.e., absent excitation. The cylindrical container was machined out of PTFE (Seal & Design Inc.) due to its low coefficients of friction.

The harvester was attached to a testing machine with servo-motors that can produce arbitrary vertical displacements. 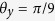 rad and 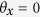 rad were then imposed to the experimental apparatus to ensure that 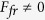. Several free fall tests (using only the moving magnet and the fixed magnet at the bottom of the container) were conducted to identify the friction coefficients. By performing a global search to find the minimum difference between experimental and simulated responses, the following coefficients were obtained: 
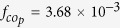
 N, 
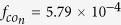
 N, 
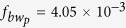
 N, 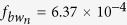 N, 
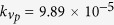
 Ns/m, 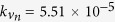 Ns/m, 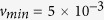 m/s. The undisturbed position of the levitated magnet was determined using both experimental tests and simulation and found to be approximately 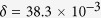 m.

**Figure 2:**
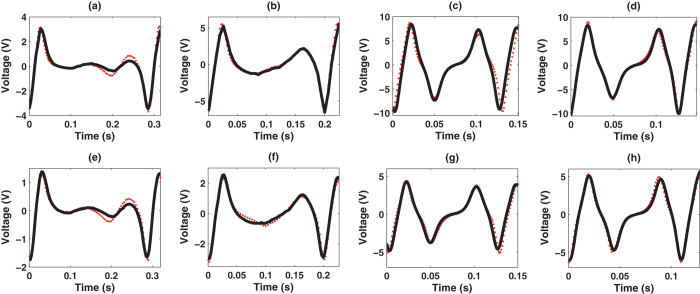
Steady-state analysis (experimental (red dots) and simulation (solid black lines)) for experiments: (a) T2; (b) T4; (c) T6; (d) T8; (e) T1; (f) T3; (g) T5; (h) T7.

**Figure 3:**
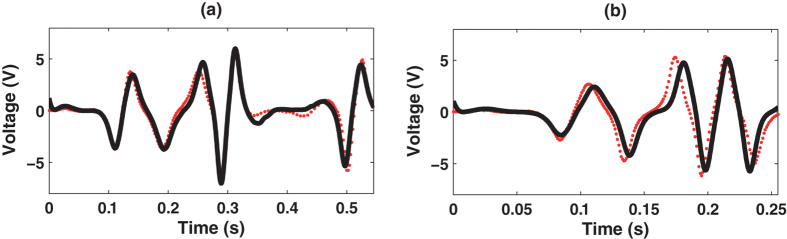
Transient analysis (experimental (red dots) and simulation (solid black lines)) for experiments: (a) T4, CC = 93.85%, EE = 7.51%; (b) T7, CC = 88.62%, EE = 6.23%.

Our system model was validated by comparing predictions with measurements with the harvester subjected to sinusoidal motion. This choice of excitation was made due to the fact that a large number of applications use cyclic motion to harvest energy. Measurements were taken for frequencies in the 3–10 Hz range. Very low voltage output was obtained for frequencies lower than 3 Hz (mean absolute voltages lower than 0.3 V for *R*_*l*_ = 89.3 kΩ and 0.15 V for *R*_*l*_ = 3.5 kΩ), and voltages exceeding the analog input range of the acquisition board (upper and lower saturation values: 10 V and −10 V) was obtained for frequencies higher than 10 Hz. The steady-state and transient responses of the energy harvester were analysed. The results of the steady-state analysis are shown in Fig. 2 and Table 2. These results demonstrate that the model accurately predicts the highly non-linear behavior of the harvester. Note that very good agreement between experimental and simulation results were achieved with energy errors lower than 14.15% (mean absolute percentage error is 6.02%) and cross-correlations higher than 86%. Similar results were obtained for the transient response, as shown in Fig. 3, i.e., the same energy errors and cross-correlations were obtained.

## Discussion

One of the main goals of this study was to develop the less complex model that could ensure very good validation results. These were achieved by neglecting: (a) the non-concentricity between the moving magnet and the fixed magnets; (b) the dynamic effects of the friction force; (c) the effects of non-axiality and non-uniformity of the friction force along the overall length of the inner wall of the container; and (d) the several components of viscous fluidic damping and air compressibility. Although further research efforts may be conducted to develop more complex models, the model presented in this paper can be used for achieving very good predictions of the energy harvesting in nonlinear regimes.

The careful selection of appropriate materials, surface finish and container architecture is also required to maximize the harvester performance. Container materials should have friction coefficients as low as possible, and with negligible magnetic permeability. PTFE is among the materials that meet such requirements. Harvester architectures using a tight-fit container-magnet interface are superior to other architectures, as they minimize the coil-magnet distance, maximizing the magnetic flux through the coils. More complex nonlinearities will most likely be observed if loose-fit container-magnet interfaces are used, due to the increasing effects of non-axiality of the moving magnet on friction force. The use of square containers and cylindrical magnets minimize the friction forces (due to the lower contact areas), but the magnetic flux through the coils would be lower. Manufacturing methods that minimize the surface roughness, ensuring similar friction coefficients along its area, also minimize nonlinearities of contact friction. Conventional machining will most likely produce worse surface finish than using chemical processes. The model proposed here can be used as a predictive tool if the requirement for harvesting performance optimization described above are taken into account. If the same materials, architectures and manufacturing methods are used, similar results will most likely be achieved.

**Figure 4:**
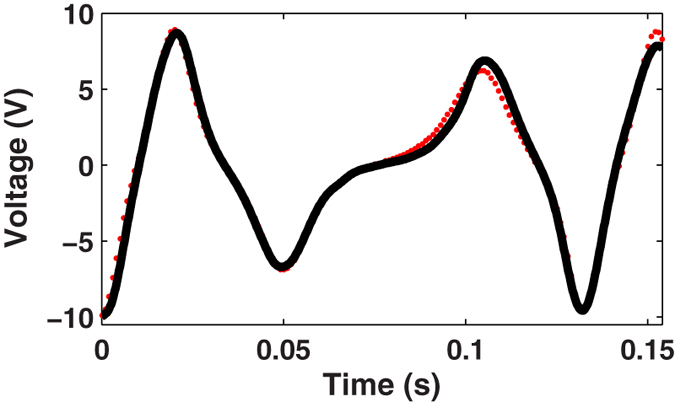
Steady-state analysis (experimental (red dots) and simulation (solid black lines)) for experiment T6 considering *M*_*m*_ = 8.1 × 10^5^ A/m and *δ* = 38.5 × 10^−3^ m: CC = 99.24%, EE = 4.55%.

EEs lower than 10% were achieved for most of the experiments. Only the T6 experiment exceeded this error range. To identify the most probable causes for this deviation, we hypothesized that very small errors in the identified model’s parameters and/or nonlinear effects of friction force not accurately modeled can result in higher energy errors for specific excitations. Considering the highly nonlinear behavior of 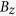, the resulting effects of using a non-optimal 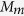 were analyzed. The steady-state analysis of the T6 experiment for *M*_*m*_ = 8.1 × 10^5^ A/m (value also within bounds reported by the manufacturer) was carried out, which change the undisturbed position of the levitated magnet in 0.2 mm (to 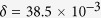 m). An energy error lower than 5% was observed, as shown in Fig. 4. Similar errors were also achieved for the other excitations analyzed in this study by applying these parameters. Hence, we can infer that non-optimal identification of the magnetization saturations can cause increasing energy errors for specific excitations. These results also suggest that a more accurate model for the friction force must be found. The proposed Karnopp friction model does not take into account the dynamic stick-slip motion of the moving magnet for PTFE-NdFeB contact surfaces and possible roughness differences that may be found along the container’s inner surface. Besides, even harvester architectures based on tight-fit container-magnet interfaces always require a time-dependent non-axiality degree of the moving magnet for levitation stability. All these effects may set differing initial conditions among experiments (the position, orientation and velocity of the moving magnet, the surface roughness, among others), mainly when successive experimental tests are conducted. We cannot also dismiss possible manifestations of chaoticity in these behaviors.

Few similarities are found between the semi-analytical model proposed here and those recently developed for diamagnetic levitation-based harvesting (DLH)^29^,^30^, namely: (a) the moving magnet is modeled as a finite set of thin current loops; (b) multilayered coil(s) are modeled as a finite set of thin circular turns; (c) the effects of the coil inductance on the current dynamics were also considered. Although DLHs are friction-free architectures, their accurate analysis requires modeling of other complex dynamics not existent in levitation systems using tight-fit containers, such as the damping forces between the moving magnet and the diamagnetic structure^29^,^30^. To our best knowledge, the proposed model that explores in more detail the fundamental transduction mechanisms of DLHs needs further improvement if it is to be used for harvester design optimization^30^. DLHs were tested only using very small excitations amplitudes^27^,^28^,^29^,^30^. Their insufficient levitation gaps have only allowed analyzing nonlinearities for very constrained motions of the moving magnet^27^,^28^,^29^,^30^. So far, stable levitation using different orientations of the harvester has been only achieved by different DLH designs^30^,^38^. However, the harvesting architecture studied in this paper and our semi-analytical model enables the analysis of energy transduction nonlinearities for wide range of motion amplitudes of the moving magnet, as well as for arbitrary orientations of the harvester.

The solver used in this work is considered one of the best global fixed-step solvers for physical systems, but it can be computationally more intensive than other solvers. Still, computing time efficiency is well demonstrated by results expressed in Table 2. Computational efficiency could be further improved if the algorithm was fully implemented in compiled code and run in dedicated computing platforms. Better selection of the fixed-step size could also improve the simulation time. The increase in computational efficiency compared to FEM was not quantified because, to our knowledge, the time to simulate FEM-based models has not yet been reported.

For known and narrowband excitations, the harvester’s characteristics described in Table 1 can be optimized prior to fabrication. This model can be used to optimize the self-powering capability of a broad range of technologies, including those that impose hard dimensional constraints, unconstrained motion amplitudes and arbitrary orientations of the harvester. Intelligent levitation-based broadband harvesting can also be achieved using such an accurate model. Intelligent control algorithms can be developed to find the most suitable positions of the fixed magnets and coil(s) for each narrowband of amplitude and frequency. It should be noted that the governing equations presented for this levitation-based harvester can also be used to model many other motion-driven electromagnetic energy harvesters, as well as to maximize the energy they can be harvested for narrow or broadband excitations. They can also be used to develop models for arbitrary tri-dimensional trajectories of harvesters.

## Methods

The hollow cylindrical structure of the harvester was machined by conventional technology. The harvester output voltage was monitored and servo-motors were controlled by a DSP board (DS1102 from dSPACE). I/O modules of DS1102 were initialized and configured in Simulink R13 (v. 5.0, Mathworks) by the Real Time Workshop (v. 5.0, Mathworks) and Real Time Interface (v. 4.4, dSPACE). An application was developed in ControlDesk (v. 2.3, dSPACE) to interact with the real-time application and to control the experiments. *V* was computed by discretizing 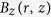 (incremental steps equal to the wire diameter) and, then, numerically integrating the resulting mesh. The 1st-order Bessel function 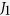 was computed as proposed by Deun and Cools^39^. In order to improve the computational efficiency, 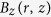, 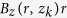, 
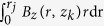
, 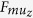 and 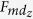 were stored in 3D Look-up Tables. Equations (7) and (9) were compiled by using Matlab S-Functions API.

The simulations were carried out using Simulink R2014a (v. 8.3, Mathworks). Equations (8) and (18) were solved via numerical integration using global Simulink fixed-step solver ode14x (fixed-step size: 2.5 ms; solver jacobian method: ‘full perturbation’; extrapolation order: 4) so that high solution accuracy and computational efficiency could be achieved. A global search algorithm from Matlab R2014a (‘GlobalSearch’ solver; ‘fmincon’ to find the minimum of the constrained squared error function) was used to find the friction coefficients. The same algorithm was used to compute the saturation magnetizations of magnets: firstly, the force between the moving magnet and each of the fixed magnets were experimentally determined within the interval of possible distances between the two faces of the magnets (0.1 to 50 mm); finally, the matching of the experimental measurements to the theoretical prediction given by Eq. 10 was carried out (the sets of lower and upper bounds of saturation magnetizations reported by the manufacturer were considered in the constrained optimization).

The energy errors and cross-correlations were computed using the following equations^40^:


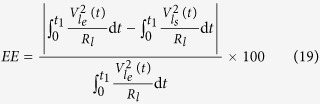



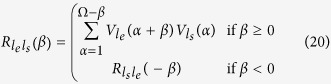



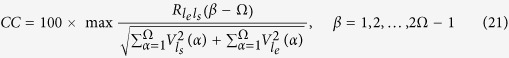


where 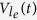 and 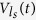 are respectively the experimental and simulated voltage harvested on the load 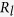 in the time domain; 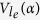 and 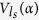 are the discrete counterparts of 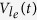 and 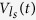 (length: Ω; 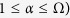; 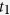 is the end of the cycle time. The trapezoidal numerical integration was used to compute Eq. 19.

## Additional Information

**How to cite this article**: Santos, M. P. S. *et al.* Magnetic levitation-based electromagnetic energy harvesting: a semi-analytical non-linear model for energy transduction. *Sci. Rep.* **6**, 18579; doi: 10.1038/srep18579 (2016).

## Figures and Tables

**Table 1:** Harvester’s characteristics.

Characteristics	Value	Units
*l*_*m*_	58 × 10^−3^	m
*l*_*c*_	20 × 10^−3^	m
*l*_*i*_	23.5 × 10^−3^	m
*d*_*h*_	16 × 10^−3^	m
*d*_*c*_	8.2 × 10^−3^	m
*d*_*m*_	6.2 × 10^−3^	m
*R*_*m*_	3 × 10^−3^	m
*R*_*u*_	3 × 10^−3^	m
*R*_*d*_	3 × 10^−3^	m
*L*_*m*_	6 × 10^−3^	m
*L*_*u*_	1 × 10^−3^	m
*L*_*d*_	1 × 10^−3^	m
*M*_*m*_	8 × 10^5^	A/m
*M*_*u*_	7.61 × 10^5^	A/m
*M*_*d*_	7.61 × 10^5^	A/m
*m*	1.24 × 10^−3^	kg
*N*_*r*_*N*_*z*_	15000	—
Coil wire diameter	6.8 × 10^−5^	m
*R*_*w*_	3.63	kΩ
*L*_*w*_	1.009	H
*R*_*l*_	89.3|3.5	kΩ

**Table 2:** Validation results^a^,^b^.

Exp.	*P*_*z*_ (mm)^c^	*R*_*l*_ (kΩ)	CC (%)	EE (%)	ST (sec)^d^
T1	17 *sin* (7*πt*)	3.5	86.20	9.31	≈12.5
T2	17 *sin* (7*πt*)	89.3	88.30	4.26	≈12.4
T3	12.25 *sin* (10*πt*)	3.5	98.24	6.38	≈9.3
T4	12.25 *sin* (10*πt*)	89.3	88.03	2.46	≈9.1
T5	7.75 *sin* (15*πt*)	3.5	92.78	0.59	≈5.8
T6	7.75 *sin* (15*πt*)	89.3	90.63	14.15	≈5.9
T7	6 *sin* (18*πt*)	3.5	88.53	4.72	≈5.1
T8	7 *sin* (16*πt*)	89.3	94.79	6.27	≈5.9

^a^Abbreviations: CC - Cross-correlation; EE - Energy error; ST - Simulation time.

^b^Results are referred to a cycle in steady state responses.

^c^*P*_*x*_ = 0 m, *P*_*y*_ = 0 m, *θ*_*x*_ = 0 rad, *θ*_*y*_ = *π*/9 rad.

^d^2.5 GHz CPU, 8 GB RAM, Windows 7 operating system.
